# No relationship between 2',3'-cyclic nucleotide 3'-phosphodiesterase and schizophrenia in the Chinese Han population: an expression study and meta-analysis

**DOI:** 10.1186/1471-2350-10-31

**Published:** 2009-04-06

**Authors:** Ronglin Che, Wei Tang, Jing Zhang, Zhiyun Wei, Zhao Zhang, Ke Huang, Xinzhi Zhao, Jianjun Gao, Guoquan Zhou, Peirong Huang, Lin He, Yongyong Shi

**Affiliations:** 1 Bio-X Center, Key Laboratory for the Genetics of Developmental and Neuropsychiatric Disorders (Ministry of Education), Shanghai Jiao Tong University, Shanghai 200030, PR China; 2 Institutes of Biomedical Sciences, Fudan University, 130 Dongan Road, Shanghai 200032, PR China; 3 Institute of Neuropsychiatric Science and Metabonomics, Changning Institute of Mental Health, the Bio-X Center Hospital, Shanghai, PR China; 4 Institute for Nutritional Sciences, Shanghai Institute of Biological Sciences, Chinese Academy of Sciences, Shanghai, PR China

## Abstract

**Background:**

2',3'-Cyclic nucleotide 3'-phosphodiesterase (*CNP*), one of the promising candidate genes for schizophrenia, plays a key part in the oligodendrocyte function and in myelination. The present study aims to investigate the relationship between *CNP *and schizophrenia in the Chinese population and the effect of different factors on the expression level of *CNP *in schizophrenia.

**Methods:**

Five *CNP *single nucleotide polymorphisms (SNPs) were investigated in a Chinese Han schizophrenia case-control sample set (n = 180) using direct sequencing. The results were included in the following meta-analysis. Quantitative real-time polymerase chain reaction (PCR) was conducted to examine *CNP *expression levels in peripheral blood lymphocytes.

**Results:**

Factors including gender, genotype, sub-diagnosis and antipsychotics-treatment were found not to contribute to the expression regulation of the *CNP *gene in schizophrenia. Our meta-analysis produced similar negative results.

**Conclusion:**

The results suggest that the *CNP *gene may not be involved in the etiology and pathology of schizophrenia in the Chinese population.

## Background

There is accumulating evidence pointing to abnormalities in oligodendrocyte function and myelination as critical factors in the etiology and pathology of schizophrenia[1,2]. Myelination and factors that affect myelination, such as the oligodendroglia function, are critical processes that could profoundly affect neuronal connectivity, especially given the diffuse distribution of oligodendrocytes and the widespread distribution of brain regions that have been implicated in schizophrenia. A series of micro-array studies have indicated significantly reduced expression levels of oligodendrocyte and myelin-related genes in the brains of schizophrenics compared with unaffected controls [3-7].

2',3'-Cyclic nucleotide 3'-phosphodiesterase (*CNP*) is used as a marker protein of myelin-forming glial cells. In brain development, *CNP *is distributed in cells of the oligodendrocyte lineage and is maintained throughout life [8]. Lower expression levels of *CNP *have been detected in the postmortem brains of schizophrenic patients[3,7,9]. Hakak et al. used an expression microarray in the postmortem dorsolateral prefrontal cortex of schizophrenics and controls and detected notable differential expression of myelination-related genes, suggesting a disruption oligodendrocyte function in schizophrenics[3]. Tkachev et al. found that the brains of schizophrenia and bipolar patients showed down-regulation of key oligodendrocyte and myelination genes, as well as of transcription factors that regulate these genes, compared with control brains[9]. In post-mortem studies of the anterior frontal cortex Flynn et al. found lower immunoreactivity of protein encoded by the *CNP *gene in schizophrenia patients (*P *= 0.05)[10]. In a case-control study Peirce et al. identified significant association between the exonic SNP rs2070106 and *CNP *expression (*P *< 0.001) and lower expression levels of the A allele (*P *= 0.04) in white subjects from the United Kingdom and Ireland [11]. *CNP *maps to 17q21.2, a region which shows strong evidence for linkage with schizophrenia as indicated by a study in a single pedigree (logarithm odds score = 8.32, genomewide *P *< 0.02) [12]. Byne et al. found *CNP *to be more highly expressed in females than males across all nuclei, suggesting that other factors such as gender may be involved in oligodendrocyte functions linked to schizophrenia[13].

A number of studies have recently been carried out on transgenic mice to clarify the role of *CNP*. Lappe-Siefke et al. showed that *CNP*-deficient mice displayed a reduction in overall brain size, enlarged ventricles and corpus callosum atrophy, features which were also observed in schizophrenia patients[8]. Rasband et al. reported that *CNP*-null mice exhibited disrupted axon-glia interactions in the central nervous system, factors which may also be implicated in schizophrenia[14].

To further investigate the role of the *CNP *gene locus in schizophrenia susceptibility, we genotyped five SNPs (rs4796750, rs8078650, rs2070106, rs11079028 and rs4796751) and performed quantitative real-time PCR to determine the factors associated with gene expression and a meta-analysis to investigate association between the *CNP *gene and schizophrenia.

## Methods

### Subjects

A total of 86 unrelated schizophrenia patients (29 males and 57 females with a mean age of 53.8 years, SD = 11.5), and 94 control individuals (41 males and 53 females with a mean age of 51.9 years, SD = 11.3) were tested for the expression study. The average onset age of disease was 26.6, SD = 10.4. Cases and controls underwent a clinical interview administered by two independent senior psychiatrists, based on Diagnostic and Statistical Manual of Mental Disorders, version IV (DSM-IV) (American Psychiatric Association). Those identified as schizophrenia patients were then diagnosed for inclusion in subgroups as follows: undifferentiated (n = 62), paranoid (n = 18), disorganized (n = 3), catatonia (n = 2), residual (n = 1). No structural diagnostic interview method was used. All the cases were hospitalized and recruited from the Changning Institute of Mental Health (the Bio-X Center Hospital) in Shanghai, East China. The controls were drawn from the general population in Shanghai. None had a history of psychotic disorders. All subjects were Chinese Han in origin. A written informed consent for the study, reviewed and approved by the Shanghai Ethics Committee of Human Genetic Resources, was obtained from all participants.

All patients were subjected to a washout period of more than eight weeks and then treated with a single antipsychotic chosen according to individual clinical assessment as follows: 43 patients with chlorpromazine at a daily dose ranging from 100 to 500 mg/d, 4 patients with risperidone from 0.5 to 3 mg/d, 11 patients with aripiprazole from 10 to 20 mg/d, and 23 patients with clozapine from 25 to 250 mg/d. The dosage was varied in individual cases where there was intolerance to the maximum dosage.

### Genotyping

Genomic DNA was extracted from venous blood using a modified phenol/chloroform method. For SNP selection, we used the HapMap database  and included five SNPs (rs4796750, rs8078650, rs2070106, rs11079028 and rs4796751) from the Chinese section. PCR amplifications of these five SNPs in the *CNP *gene were first performed for all subjects on the GeneAmp PCR 9700 System and then genotyped using direct sequencing on an ABI 3100 genetic analyzer using the BigDye Terminator Cycle Sequencing Kit (Applied Biosystems).

### RNA Extraction and cDNA Synthesis

Total RNA was extracted using Trizol reagent. RNA integrity was confirmed by direct visualization of 18S and 28S rRNA bands after agarose-gel electrophoresis. RNA samples were reverse transcribed using the SuperScript first-strand synthesis system (Invitrogen) and random hexamers.

### Real-Time Quantitative PCR Expression Assay

We performed real-time PCR on the ABI 7900 system (Applied Biosystems). Reactions were performed in a 10-ul volume including diluted cDNA samples, primers, and SYBR Green I Mastermix (Applied Biosystems). We collected real-time PCR data using SDS software (version 2.1 [Applied Biosystems]). Both beta-actin (*ACTB*) and *CNP *were tested four times for each sample.

### Statistical Analysis

#### Case-control study

Allele and genotype frequencies were calculated using the online software SHEsis [15]. The pairwise linkage disequilibrium (LD) and haplotype analysis were conducted on Haploview software version 3.11 (available at ). Group comparisons for the effect of gender, genotype, subtype, and specific drug on the expression level of *CNP *were analyzed using a t test and one-way ANOVA test on SPSS for Windows, version 15.0.

#### Meta-analysis

Eligible studies had to meet all of the following criteria: (1) they were published in peer-reviewed journals, (2) they were independent studies using original data, (3) they provided sufficient data to calculate the odds ratio (OR) with confidence interval (CI) and P-value, (4) they were case-control association studies investigating *CNP *polymorphisms, (5) they described the relevant genotyping primers, machines and protocols or provided reference to them, (6) they diagnosed schizophrenic patients according to DSM-IV criteria, and (7) they used healthy individuals as controls. We searched PubMed citations  up to January 2009 using keywords "*CNP*" and "schizophrenia". A Cochran's *X*^2^-based Q statistical test was conducted to assess heterogeneity and thus to ensure that each group of studies was suitable for meta-analysis. When heterogeneity was detected, the random effects model was adopted; otherwise, the fixed effects model was used. We assessed publication bias using an ancillary procedure for funnel plot asymmetry, as described by Egger et al[16]. The significance of the pooled odds ratios was determined by the Z-test. The analysis was performed using Comprehensive Meta-Analysis (Version 2.2.046, BIOSTAT).

## Results

**Figure 1:**
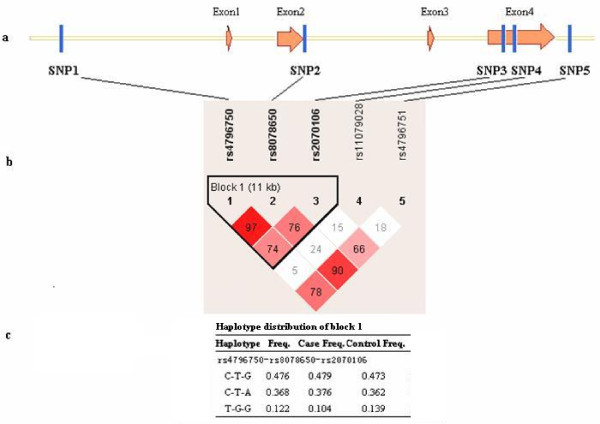
**Genomic structure, linkage disequilibrium of *CNP *and haplotype distribution**. a. Genomic structure and locations of polymorphic sites in *CNP*. b. Estimation of linkage disequilibrium (D' value) between five SNPs. c. Haplotype distribution of block1.

86 schizophrenics and 94 unaffected controls were genotyped and frequencies were calculated (Table 1). There was evidence of linkage disequilibrium (LD; D'>0.7) between rs4796750, rs8078650 and rs2070106, which were considered to reside within the same haplotype block (Fig 1b). In this block three common haplotypes accounted for 97% of the variation (Fig 1c).

**Table 1:** Allele and genotype distribution of SNPs in *CNP *in schizophrenia patients (case) and control subjects

**SNP**	**Sample**	**Allele(Freq.)**		**Genotype(Freq.)**		
		C	T	C/C	C/T	T/T
SNP1	Case	148(0.871)	22(0.129)	65(0.765)	18(0.212)	2(0.024)
rs4796750	Control	154(0.846)	28(0.154)	65(0.714)	24(0.264)	2(0.022)
		T	G	T/T	T/G	G/G
SNP2	Case	147(0.865)	23(0.135)	65(0.765)	17(0.200)	3(0.035)
rs8078650	Control	155(0.842)	29(0.158)	66(0.717)	23(0.250)	3(0.033)
		A	G	A/A	A/G	G/G
SNP3	Case	66(0.393)	102(0.607)	15(0.179)	36(0.429)	33(0.393)
rs2070106	Control	68(0.370)	116(0.630)	14(0.152)	40(0.435)	38(0.413)
		C	T	C/C	C/T	T/T
SNP4	Case	101(0.616)	63(0.384)	28(0.341)	45(0.549)	9(0.110)
rs11079028	Control	103(0.579)	75(0.421)	28(0.315)	47(0.528)	14(0.157)
		T	C	T/T	T/C	C/C
SNP5	Case	25(0.145)	147(0.855)	3(0.035)	19(0.221)	64(0.744)
rs4796751	Control	25(0.133)	163(0.867)	3(0.032)	19(0.202)	72(0.766)

**Figure 2:**
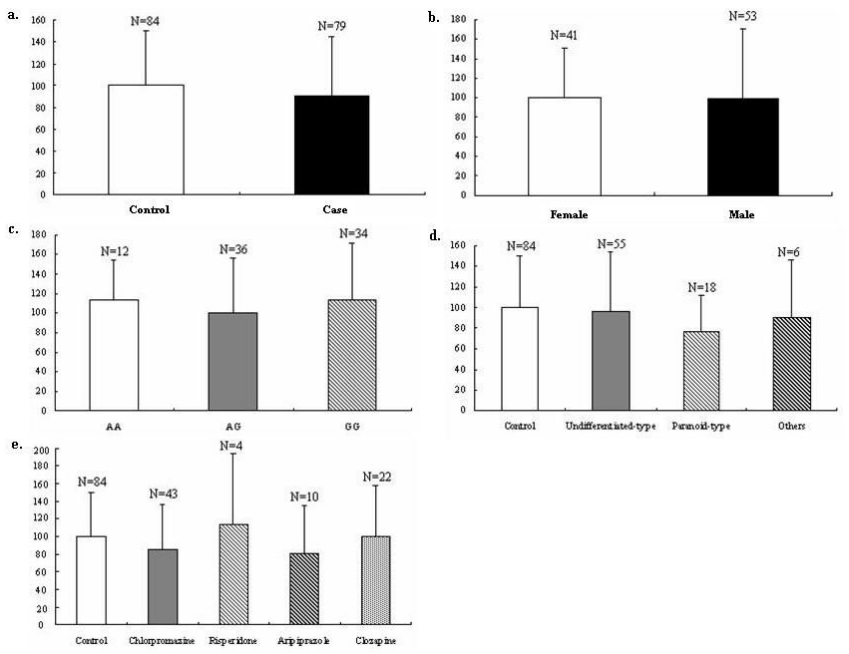
**Expression analyses of *CNP***. **a**. Expression level of *CNP *in the PBLs from unaffected controls and patients with schizophrenia. The average level of *CNP *in controls was defined as 100. No significant difference between controls and cases. **b**. Expression level of *CNP *from unaffected controls between females and males. The average level of *CNP *in females was defined as 100. No significant difference between females and males. **c**. Expression level of *CNP *from unaffected controls in the SNP rs2070106. The average level of *CNP *in AG was defined as 100. No significant difference among genotypes. **d**. Expression level of *CNP *from patients characterized with subtypes. The average level of *CNP *in controls was defined as 100. No significant difference among these subtypes. **e**. Expression level of *CNP *from patients treated with four separate antipsychotic drugs. The average level of *CNP *in controls was defined as 100. No significant difference among these subgroups.

Real-time PCR was then performed to quantify the expression level of *CNP *in the peripheral blood lymphocytes (PBLs). Some results were discarded on the basis of showing deviation after four replications. We obtained valid expression data for 79 schizophrenics and 84 controls. As shown in Fig 2a, the expression of *CNP *was reduced by 10% in the 79 schizophrenics compared with the 84 controls, but the difference was not significant. To exclude complicating factors among patients, we compared expression levels in males and females using unaffected controls (Fig 2b), with respect to three genotypes of rs2070106 (Fig 2c). With regard to the schizophrenia subtypes, we compared the expression levels of the undifferentiated-type, the paranoid-type and other types (consisting of 3 disorganized-type, 2 catatonic-type and 1 residual-type) as presented in Fig 2d. Fig 2e shows the effects on the expression of *CNP *of the four antipsychotics used, namely, chlorpromazine, risperidone, aripiprazole and clozapine. However, no statistical difference was found in any of the above groups.

Four independent studies meeting the selection criteria were included in the meta-analysis investigating association between *CNP *gene and schizophrenia[11,17,18]. The OR with 95% CIs for the case-control studies are shown in Table 2. No statistically significant association was found for rs2070106 and rs8078650 in the allelic analysis. No publication bias was found.

**Table 2:** Meta-analysis of the combined case-control studies for rs2070106 and rs8078650 in *CNP*

**SNP ID**	**Studies**	**Ethnicities**	**Category**	**No.**	**MAF^a^**	**OR(95% CI)**
rs2070106	Present study	Han Chinese	Case	84	0.393	1.10(0.60,2.03)
			Control	92	0.370	
	Tang et al.(2007)	Han Chinese	Case	426	0.403	1.08(0.82,1.42)
			Control	437	0.384	
	Peirce et al.(2006)	UK&Ireland White	Case	683	0.343	1.18(0.94,1.48)
			Control	693	0.306	
	Usui et al.(2006)	Japanese	Case	759	0.38	1.00(0.81,1.23)
			Control	729	0.38	
	Pooled	All		3903		1.08(0.95,1.23)
rs8078650	Present study	Han Chinese	Case	85	0.135	1.20(0.52,2.78)
			Control	92	0.158	
	Tang et al.(2007)	Han Chinese	Case	422	0.146	1.07(0.74,1.56)
			Control	436	0.155	
	Usui et al.(2006)	Japanese	Case	746	0.14	1.00(0.75,1.34)
			Control	745	0.14	
	Pooled	All		2526		1.04(0.83,1.30)

^a^: Minor allele frequency

## Discussion

Previous case-control studies in this area have been inconsistent in their findings. Peirce et al. reported that the A allele of rs2070106 was significantly associated with schizophrenia (P = 0.04) in the white populations of the United Kingdom and Ireland [11]. However, Japanese[17] and Chinese[18] studies showed no significant association. In a recent family-based association study, Voineskos reported the rs2070106 risk allele G as being associated with schizophrenia in a Caucasian population[19]. Genetic heterogeneity may account for the inconsistency among the above studies, as they are based on different ethnic populations[20,21]. The LD block structure of *CNP *varies between different populations according to the HapMap database  and other reports. No strong LD was observed in the population of western European ancestry (CEU) while a tight LD block was identified in the Japanese and Chinese populations. Additionally, we found that the allele frequency distribution of SNPs with *CNP *varied across different populations according to the HapMap database. For example, the rs2070106 A allele frequency is 32.2% in Asians but 40.8% in Europeans, while the rs8078650 G allele frequency is 22.2% in Chinese subjects but 13.3% in the Japanese population. It has been reported that the power to detect marginal genetic effects may be influenced by variations in allele frequencies across populations [22].

We used peripheral blood lymphocytes to investigate gene expression of *CNP*. Blood lymphocytes can serve as a convenient and accessible probe to detect cellular function of the brain, including gene expression[23]. Critical pathways in schizophrenia can be studied in peripheral tissue as part of the strategy in analyzing functional genomic convergence[24]. Previous reports on schizophrenia have suggested that altered expression of genes associated with myelination of neurons in peripheral blood lymphocytes is consistent with results from postmortem brain tissue studies [25]. Our results suggest that expression levels of *CNP *in schizophrenic patients are lower by only 10% compared to unaffected controls, an insignificant difference. One previous study identified higher *CNP *mRNA expression in females than in males in all thalamic regions (*P *= 0.0014)[13]. However, our own study detected no difference between females and males either in the healthy subjects alone or in the combined samples. Iwamoto et al. reported that the expression of *CNP *transcript was associated with the rs2070106 genotype in the brains of control subjects[26]. Yet, our study did not replicate this finding with regard to PBLs.

To date, no publication has reported the effect on *CNP *expression based on different schizophrenic subtypes and different drug treatments. Our data showed no statistically significant differences with respect to two major diagnosis subtypes and the four antipsychotic-treating subgroups. However, we need to be cautious in drawing this conclusion because the sample size is relatively small. The different gender distribution between patients and controls also presents a potential complication. The effect of these factors on *CNP *expression requires further study.

## Conclusion

To sum up, our expression assay did not support a relationship between *CNP *and schizophrenia in the Chinese population. Similarly, the meta-analysis demonstrated no significant association between the two polymorphisms (rs2070106 and rs8078650) and schizophrenia. Ethnic background is a factor to be considered in further functional research on oligodendrocyte- and myelin-related pathways and their roles in the pathology and etiology of psychiatric disorders.

## Competing interests

The authors declare that they have no competing interests.

## Authors' contributions

RC and WT designed the study carried out the experiments and data analysis. RC undertook the statistical analysis and wrote the first draft of the manuscript. All authors contributed to the sample recruitment and have approved the final manuscript.

## Pre-publication history

The pre-publication history for this paper can be accessed here:



## References

[B1] Hof PR, Haroutunian V, Copland C, Davis KL, Buxbaum JD (2002). Molecular and cellular evidence for an oligodendrocyte abnormality in schizophrenia. Neurochem Res.

[B2] Davis KL, Haroutunian V (2003). Global expression-profiling studies and oligodendrocyte dysfunction in schizophrenia and bipolar disorder. Lancet.

[B3] Hakak Y, Walker JR, Li C, Wong WH, Davis KL, Buxbaum JD, Haroutunian V, Fienberg AA (2001). Genome-wide expression analysis reveals dysregulation of myelination-related genes in chronic schizophrenia. Proc Natl Acad Sci USA.

[B4] Katsel P, Davis KL, Haroutunian V (2005). Variations in myelin and oligodendrocyte-related gene expression across multiple brain regions in schizophrenia: a gene ontology study. Schizophr Res.

[B5] Haroutunian V, Katsel P, Dracheva S, Stewart DG, Davis KL (2007). Variations in oligodendrocyte-related gene expression across multiple cortical regions: implications for the pathophysiology of schizophrenia. Int J Neuropsychopharmacol.

[B6] Aston C, Jiang L, Sokolov BP (2004). Microarray analysis of postmortem temporal cortex from patients with schizophrenia. J Neurosci Res.

[B7] Davis KL, Stewart DG, Friedman JI, Buchsbaum M, Harvey PD, Hof PR, Buxbaum J, Haroutunian V (2003). White matter changes in schizophrenia: evidence for myelin-related dysfunction. Arch Gen Psychiatry.

[B8] Lappe-Siefke C, Goebbels S, Gravel M, Nicksch E, Lee J, Braun PE, Griffiths IR, Nave KA (2003). Disruption of Cnp1 uncouples oligodendroglial functions in axonal support and myelination. Nat Genet.

[B9] Tkachev D, Mimmack ML, Ryan MM, Wayland M, Freeman T, Jones PB, Starkey M, Webster MJ, Yolken RH, Bahn S (2003). Oligodendrocyte dysfunction in schizophrenia and bipolar disorder. Lancet.

[B10] Flynn SW, Lang DJ, Mackay AL, Goghari V, Vavasour IM, Whittall KP, Smith GN, Arango V, Mann JJ, Dwork AJ (2003). Abnormalities of myelination in schizophrenia detected in vivo with MRI, and post-mortem with analysis of oligodendrocyte proteins. Mol Psychiatry.

[B11] Peirce TR, Bray NJ, Williams NM, Norton N, Moskvina V, Preece A, Haroutunian V, Buxbaum JD, Owen MJ, O'Donovan MC (2006). Convergent evidence for 2',3'-cyclic nucleotide 3'-phosphodiesterase as a possible susceptibility gene for schizophrenia. Arch Gen Psychiatry.

[B12] Williams NM, Norton N, Williams H, Ekholm B, Hamshere ML, Lindblom Y, Chowdari KV, Cardno AG, Zammit S, Jones LA (2003). A systematic genomewide linkage study in 353 sib pairs with schizophrenia. Am J Hum Genet.

[B13] Byne W, Dracheva S, Chin B, Schmeidler JM, Davis KL, Haroutunian V (2008). Schizophrenia and sex associated differences in the expression of neuronal and oligodendrocyte-specific genes in individual thalamic nuclei. Schizophr Res.

[B14] Rasband MN, Tayler J, Kaga Y, Yang Y, Lappe-Siefke C, Nave KA, Bansal R (2005). CNP is required for maintenance of axon-glia interactions at nodes of Ranvier in the CNS. Glia.

[B15] Shi YY, He L (2005). SHEsis, a powerful software platform for analyses of linkage disequilibrium, haplotype construction, and genetic association at polymorphism loci. Cell Res.

[B16] Egger M, Davey Smith G, Schneider M, Minder C (1997). Bias in meta-analysis detected by a simple, graphical test. Bmj.

[B17] Usui H, Takahashi N, Saito S, Ishihara R, Aoyama N, Ikeda M, Suzuki T, Kitajima T, Yamanouchi Y, Kinoshita Y (2006). The 2',3'-cyclic nucleotide 3'-phosphodiesterase and oligodendrocyte lineage transcription factor 2 genes do not appear to be associated with schizophrenia in the Japanese population. Schizophr Res.

[B18] Tang F, Qu M, Wang L, Ruan Y, Lu T, Zhang H, Liu Z, Yue W, Zhang D (2007). Case-control association study of the 2',3'-cyclic nucleotide 3'-phosphodiesterase (CNP) gene and schizophrenia in the Han Chinese population. Neurosci Lett.

[B19] Voineskos AN, de Luca V, Bulgin NL, van Adrichem Q, Shaikh S, Lang DJ, Honer WG, Kennedy JL (2008). A family-based association study of the myelin-associated glycoprotein and 2',3'-cyclic nucleotide 3'-phosphodiesterase genes with schizophrenia. Psychiatr Genet.

[B20] Colhoun HM, McKeigue PM, Davey Smith G (2003). Problems of reporting genetic associations with complex outcomes. Lancet.

[B21] Pulver AE, Mulle J, Nestadt G, Swartz KL, Blouin JL, Dombroski B, Liang KY, Housman DE, Kazazian HH, Antonarakis SE (2000). Genetic heterogeneity in schizophrenia: stratification of genome scan data using co-segregating related phenotypes. Mol Psychiatry.

[B22] Marchini J, Donnelly P, Cardon LR (2005). Genome-wide strategies for detecting multiple loci that influence complex diseases. Nat Genet.

[B23] Gladkevich A, Kauffman HF, Korf J (2004). Lymphocytes as a neural probe: potential for studying psychiatric disorders. Prog Neuropsychopharmacol Biol Psychiatry.

[B24] Vawter MP, Ferran E, Galke B, Cooper K, Bunney WE, Byerley W (2004). Microarray screening of lymphocyte gene expression differences in a multiplex schizophrenia pedigree. Schizophr Res.

[B25] Bowden NA, Weidenhofer J, Scott RJ, Schall U, Todd J, Michie PT, Tooney PA (2006). Preliminary investigation of gene expression profiles in peripheral blood lymphocytes in schizophrenia. Schizophr Res.

[B26] Iwamoto K, Ueda J, Bundo M, Nakano Y, Kato T (2008). Effect of a functional single nucleotide polymorphism in the 2',3'-cyclic nucleotide 3'-phosphodiesterase gene on the expression of oligodendrocyte-related genes in schizophrenia. Psychiatry Clin Neurosci.

